# Evaluation of the relationships between beta-human chorionic gonadotropin (β-hCG) blood levels and pregnancy: A retrospective study

**DOI:** 10.12669/pjms.41.7.12250

**Published:** 2025-07

**Authors:** Olgun Goktas

**Affiliations:** Dr. Olgun Goktas, Associate Professor, Uludag University Family Health Center, 16059, Gorukle Campus-Nilufer, Bursa, Turkey.

**Keywords:** β-human chorionic gonadotropin, complications, diseases, pregnancy, primary care

## Abstract

**Objective:**

To retrospectively evaluate the relationships between beta-human chorionic gonadotropin (β-hCG) blood levels and pregnancy in primary care.

**Methods:**

The retrospective study was conducted in Bursa Uludağ University Family Health Center in Turkey between October 1^st^ and 31st October 2023. In the study, the data of pregnant women who applied to the Family Health Center for pregnancy examinations and follow-ups within the five years between January 1, 2018, and December 31, 2022, were taken from the database and evaluated retrospectively. History of current and previous pregnancies, sociodemographic findings, current diseases, allergies, cancer, genetic disease, Rh incompatibility, medication use, pregnancy risk factors, pregnancy complications, and delivery method and outcomes were obtained. Blood total β-hCG measurement results were examined for pregnancy and differential diagnoses. P-values below 0.05 were considered statistically significant. Analyses were made in the SPSS 25.0 program.

**Results:**

The mean age of the 239 pregnant women who participated in the study was 28.74±4.02, and the mean total gestational age was 37.28±7.68 weeks. It was determined that 96.2% of the pregnant women had total β-hCG levels in the blood above 5 mIU/ml. Risk factors were present in 26.4% of the pregnant women, and 3.8% of them developed complications during pregnancy. Blood β-hCG levels were higher in those who had an additional disease during pregnancy, those who were on medication, those who had risk factors, and those who developed complications. While total β-hCG measurement levels were lower in those who had normal or cesarean live births, they were higher in those who had abortions (p=0.03).

**Conclusion:**

In our study, blood β-hCG levels were higher in pregnant women who had an additional disease, those who were on medication, those who had risk factors and those who developed complications. Total β-hCG measurement levels were lower in those who had normal or cesarean live births, while they were higher in those who had abortions. Different results in blood β-hCG levels require a holistic approach to evaluate pregnancy. In this critical process, the collaboration of family physicians with obstetrics, gynecology and other clinics is important.

## INTRODUCTION

The Human chorionic gonadotropin (hCG) is a major hormone secreted by the trophoblast in early development. hCG consists of four main isoforms: Classical hCG, hyperglycosylated hCG, free subunit, and sulfated hCG. It has two subunits, alpha and beta. hCG hormone has several actions aimed at protecting the embryo, including maintaining progesterone secretion by the corpus luteum. It can be detected in maternal serum as early as eight days after conception. The serum level, produced and secreted by the syncytial trophoblast, represents the trophoblastic mass. The hCG level increases dynamically during early pregnancy. Low serum hCG levels in early pregnancy have been reported as an indicator of adverse pregnancy outcomes, such as chemical and ectopic pregnancies, as well as spontaneous abortion.^1^-^3^ Early pregnancy can be diagnosed with the combined use of hCG and transvaginal ultrasound. However, their incorrect use and especially their misinterpretation can lead to incorrect information and results. Therefore, hCG dynamics should be well known, especially in early pregnancy, and the process should be managed with appropriate approaches.^4^

In a study evaluating the relationships between serum β-human chorionic gonadotropin levels and early pregnancy outcomes, it was reported that high serum β-hCG levels led to an increase in multiple pregnancy rates and a decrease in biochemical pregnancy rates and ectopic pregnancy rates. It is emphasized that serum β-hCG levels predict biochemical/clinical pregnancy and single/multiple pregnancies with strong sensitivity and specificity,^5^ β-hCG levels are important as a predictive marker in terms of diagnosis and treatment in conditions affecting mortality and morbidity, such as anembryonic pregnancies in early pregnancy losses,^6^ and placenta previa, where the placenta is incorrectly placed.^7^ hCG is used as a biomarker in pregnancy diagnosis and the determination of trophoblastic and non-trophoblastic cancers and is usually a combination of antibodies, one of which is specific to the C-terminal peptide of the β-subunit.^8^

Free β-hCG is important in determining risky pregnancies in terms of both fetal and maternal outcomes. β-hCG levels < 0.2 or > 5 as MoM are considered risk factors for adverse maternal or fetal outcomes in first-trimester screening.^9^ A meta-analysis study reported that high β-hCG levels during pregnancy are associated with a high incidence of pregnancy complications and adverse pregnancy outcomes. Closer monitoring of pregnant women with high β-hCG levels and timely medical interventions to reduce the incidence of pregnancy complications and adverse outcomes are recommended.^10^ In cases where the human chorionic gonadotropin (hCG) test is positive without clinical pregnancy, “phantom hCG” is possible and leads to false diagnosis of malignancy, unnecessary chemotherapy, and surgical procedures.^11^ The first pregnancy tests of women considering pregnancy are usually performed in the family doctor clinic during counseling. Although urine tests were used in previous years to detect pregnancy, today, the beta human chorionic gonadotropin level (β-hCG) is usually checked with a blood test. If the β-hCG test performed for pregnancy diagnosis is positive, the patient is directed to the gynecology and obstetrics clinic for confirmation and obstetric and gynecological evaluation. Verification of β-hCG for both pregnancy and non-pregnancy reasons and monitoring its level are important in terms of possible diseases and complications during pregnancy as well as the normal pregnancy process. Although there are many different examples and reports in the literature, examining the relationships between β-hCG and abnormal conditions that may occur before and during pregnancy will make different contributions. The evaluation of registered pregnancy data with the family doctor is important in this respect.

In this study, we aimed to evaluate the relationships between the β-hCG blood test results performed before and during pregnancy in pregnant women registered in the family health center and the diseases, pregnancy outcomes, and complications during pregnancy.

## METHODS

In this retrospective study, pregnant women whose β-hCG blood results were checked in our Bursa Uludağ University Family Health Center in the five years between January 1, 2018, and December 31, 2022, and whose pregnancy was confirmed by the obstetrics and gynecology department were included. In this respect, all pregnant women who were followed up in our Family Health Center and met the study acceptance criteria were included in the study by making a full count. The history of current and previous pregnancies, sociodemographic findings, current diseases, allergies, cancer, genetic disease, Rh incompatibility, medication use, pregnancy risk factors, pregnancy complications, and delivery method and outcomes were obtained from the database (recorded in the Family Medicine Information Registration System).

In this respect, power levels were calculated according to the methods used in data analysis. In this context, it can be seen that including at least n=176 individuals in the study can represent 99% power and 0.51 effect size level. The study can be conducted with at least n=176 samples in this context. The current study was conducted with n=248 pregnant women who met the acceptance criteria in the relevant period, but the study was conducted with n=239 pregnant women whose data were valid. In this respect, it is seen that the sample has sufficient power and the effect size level is reasonably sufficient.

### Ethical approval:

The study was performed after approval from the Clinical Research Ethics Committee of Bursa Uludağ University, Faculty of Medicine (Ref. dated 2023-09-19/ decision no:2023-17/2) following the Declaration of Helsinki.

### Inclusion and exclusion criteria:

All pregnant women registered in our region during the specified date range were included in the study, while pregnant women who migrated or left the region for various reasons and those outside our region were not included.

### Statistical analysis:

In this study, descriptive statistics are presented with mean, standard deviation, frequency, and percentage values in the analysis of data. Mann-Whitney U test analysis was performed to examine the measurements made in the study according to beta hCG groups. Chi-square analysis was performed in the examination of proportional variables, and Fisher correction was applied when necessary. P values less than 0.05 were determined statistically in the study. Analyses were performed with SPSS 25.00 version. The power analysis of the study was performed using the G power 3.1 program.

## RESULTS

A total of 239 pregnant women participated in the study. It was determined that the average age of the individuals was 28.74±4.02. The average total weeks of pregnancy of the individuals was 37.28±7.68. In the study, it was seen that 0.4% of the pregnant were housewives, 39.3% were students, 4.6% were teachers, 23% were doctors, and 32.6% were from other professional groups. It was seen that 85.4% of the pregnant women were mostly university graduates. It was also observed that 90.4% of the pregnant women did not smoke, and 8.8% quit smoking during pregnancy. It was noted that 99.6% of the pregnant women did not drink alcohol at all. We also noticed that 31.8% of the participants had an additional disease. 1.7% of the pregnant women had cancer, 1.3% had allergies, and 11.7% had Rh incompatibility. About 28% of the pregnant women used medication regularly.

There are other risky pregnancy factors in 26.4% of the pregnant. Total β-hCG was found to be positive above 5-mIU/mL in 96.2% of the patients. Complications were observed in 3.8% of the pregnant. Pregnant patients completed their pregnancies with normal live birth in 63.6%, cesarean section and live birth in 29.3%, abortion in 6.3%, and stillbirth in 0.4% (Table-I). The average age of the pregnant women was found to be 28.74±4.02. Height measurements were found to be 164.68±9.10 cm, weight measurements were found to be 69.01±11.92 kg, and Body Mass Index (BMI) levels were found to be 26.64±21.79. The average gestational weeks were 37.28±7.68 (Table-II).

**Table-I:** Demographic and Clinical Features.

Feature		n	%
Occupation	Housewife	1	0.4%
	Student	94	39.3%
	Teacher	11	4.6%
	Doctor	55	23.0%
	Other	78	32.6%
Education	Pre-High School	4	1.7%
	High School	29	12.1%
	University	204	85.4%
	Postgraduate	2	0.8%
Smoking	Never Smoked	216	90.4%
	Quit During Pregnancy	21	8.8%
	Smoking	2	0.8%
Alcohol	Never Drinked Alcohol	238	99.6%
	Quit During Pregnancy	1	0.4%
	Drinking	0	0.0%
Current disease	No	163	68.2%
	Yes	76	31.8%
Genetic disease	No	239	100.0%
	Yes	0	0.0%
Cancer	No	235	98.3%
	Yes	4	1.7%
Allergy	No	236	98.7%
	Yes	3	1.3%
Rh incompatibility	No	211	88.3%
	Yes	28	11.7%
Medication	No	172	72.0%
	Yes	67	28.0%
Other pregnancy risk factors	No	176	73.6%
	Yes	63	26.4%
Total β-hCG measurement positive (>5 mIU/mL)	Total β-hCG Positive<5 mIU/mL	230	96.2%
	Total β-hCG Positive>5 mIU/mL	9	3.8%
Pregnancy complication	No	230	96.2%
	Yes	9	3.8%
Delivery method and/or outcome	Normal Live Birth	152	63.6%
	Cesarean Live Birth	70	29.3%
	Still Birth	1	0.4%
	Abortion	15	6.3%
	Other	1	0.4%

**Table-II:** Distributions of measurements.

Measurement	X±SD (mean±SD)
Age	28.74±4.02
Height	164.68±9.10
Weight	69.01±11.92
BMI (Body Mass Index)	26.64±21.79
Total Pregnancy Weeks	37.28±7.68

It was observed that the pregnant women’s profession (p=0.42), education level (p=0.83), smoking status (p=0.12), and alcohol consumption status (p=0.12) did not have a significant effect on the positive total β-hCG measurement status. It was also observed that the total β-hCG measurement rates of the participants with an additional disease were significantly higher than the group without the disease (p=0.04). It was further determined that the total β-hCG measurement rates did not differ in patients with cancer, allergy, and Rh incompatibility based on additional diseases (p>0.05). The total β-hCG measurement rates of the group using medication regularly were significantly higher than the groups not using medication (p=0.04).

However, the total β-hCG measurement rates were significantly higher in the group with other pregnancy risk factors (p=0.04) and in the group with complications during pregnancy (p=0.03). It was determined that the total β-hCG measurement positive rates differed according to pregnancy termination cases. It was further determined that the total β-hCG measurement positive rates were higher in the groups that had abortions. In the groups that had normal and live births by cesarean section, 96.1% of the total β-hCG measurements were below the level of 5 (p=0.03) (Table-III).

**Table-III:** Investigation of the features affecting the positivity levels of total β-hCG measurement.

		Total β-hCG measurement positive (>5 mIu/mL)				p
		Total β-hCG Positive <5 mIU/mL		Total β-hCG Positive >5 mIU/mL		
		n	%	n	%	
Occupation	Housewife	1	0.4%	0	0.0%	0.42
	Student	91	39.6%	3	33.3%	
	Teacher	11	4.8%	0	0.0%	
	Doctor	54	23.5%	1	11.1%	
	Other	73	31.7%	5	55.6%	
Education	Pre-High School	32	13.9%	1	11.1%	0.83
	University and above	198	86.1%	8	88.9%	
Smoking	Never Smoked	211	91.7%	5	55.6%	0.12
	Quit During Pregnancy	19	8.3%	2	22.2%	
	Smoking	0	0.0%	2	22.2%	
Alcohol	Never Drinked Alcohol	230	100.0%	8	88.9%	0.39
	Quit During Pregnancy	0	0.0%	1	11.1%	
Current disease	No	163	70.9%	0	0.0%	0.04*
	Yes	67	29.1%	9	100.0%	
Cancer	No	229	99.6%	6	66.7%	0.48
	Yes	1	0.4%	3	33.3%	
Allergy	No	228	99.1%	8	88.9%	0.83
	Yes	2	0.9%	1	11.1%	
Rh incompatibility	No	204	88.7%	7	77.8%	0.42
	Yes	26	11.3%	2	22.2%	
Medication	No	172	74.8%	0	0.0%	0.04*
	Yes	58	25.2%	9	100.0%	
Other pregnancy risk factors	No	176	76.5%	0	0.0%	0.04*
	Yes	54	23.5%	9	100.0%	
Pregnancy complication	No	225	97.8%	5	55.6%	0.03*
	Yes	5	2.2%	4	44.4%	
Delivery method and/or outcome	Normal Live Birth	152	66.1%	0	0.0%	0.03*
	Cesarean Live Birth	69	30.0%	1	11.1%	
	Still Birth	0	0.0%	1	11.1%	
	Abortion	9	3.9%	6	66.7%	
	Other	0	0.0%	1	11.1%	

**Chi-square test and Fisher correction,

* Significant difference at 0.05 level

It was found that there were no significant differences in age measurements (p=0.52), height (p=0.84) and weight measurements (p=0.86) in the groups with total β-hCG measurements above and below 5 mIU/mL. Body mass index (BMI) levels (p=0.03) and birth weeks were significantly lower in the groups with total β-hCG measurements above 5 mIU/mL (p=0.01) (Table-IV).

**Table-IV:** Analysis of Measurements Based on Total β-hCG Levels.

Measurement	Total β-hCG measurement positive (>5 mIu/mL)		p
	Total β-hCG Positive <5 mIU/mL	Total β-hCG Positive >5 mIU/mL	
	X±SD (mean±SD)	X±SD (mean±SD)	
Age	28.73±3.94	29±5.98	0.52
Height	164.56±9.21	167.89±4.88	0.84
Weight	69.04±12.05	68.11±8.7	0.86
BMI (Body Mass Index)	26.74±2.20	24.19±3.09	0.03*
Total Pregnancy Weeks	38.17±5.85	14.44±12.93	0.01*

**Mann Whitney U test,

* Significant difference at 0.05 level

## DISCUSSION

This study determined that 96.2% of pregnant women in our region had total β-hCG levels above 5 mIU/ml, 26.4% had risk factors, and 3.8% developed complications during pregnancy. Blood β-hCG levels were higher in those who had additional diseases during pregnancy, those who used medication, those who had risk factors, and those who developed complications. It is important that total β-hCG levels were lower in those who had normal or cesarean births, while they were higher in those who had abortions. The results of our study are important in terms of evaluating blood β-hCG levels in family medicine practice in pregnancy diagnosis and differential diagnosis. Family physicians should be very careful during this process and continue to cooperate with obstetrics, gynecology, and other clinics.

There are many hCG-related molecules in pregnancy serum and urine samples, such as unnicked hCG (hormone), nicked hCG, hyper- and hypoglycosylated hCG, free a subunit, and free b subunit. All of these molecule types are suitable for the detection of a normal pregnancy. Some submolecules may be particularly useful in the detection of abnormal pregnancies.^12^ To distinguish normal from abnormal pregnancies, hCG levels can be measured serially in the blood. In this case, although it is also supported by ultrasonography, the physician’s decision regarding symptoms and clinical signs is important in pregnancy diagnosis and follow-up.^13^ In a study, it was reported that although early intrauterine pregnancy structures allow lower β-hCG values with the development of ultrasonography, the results of the differential levels are higher.^14^ In another study, it was reported that serum placental growth factor (PLGF) and pregnancy-associated plasma protein A (PAPP-A) are effective together with β-hCG in the detection of early pregnancy. However, it is still recommended to strengthen the diagnosis with an ultrasound.^15^

It has been reported that the β-hCG level returned to normal after surgical removal of a perivascular epithelioid cell tumor detected in a postmenopausal vaginal bleeding patient with high β-hCG levels.^16^ In our study, there was no significant difference in terms of age measurements in the groups with low or high total β-hCG levels. Paraneoplastic hyperthyroidism^17^ caused by increased β-hCG due to chemotherapy treatment in a patient with a tumor, and high β-hCG levels due to choriocarcinoma^18^ have been reported. Knowing the high serum hCG level in twin pregnancy preeclampsia,^19^ 10-14 weeks pregnancy preeclampsia^20^ and 3rd trimester preeclampsia^21^ is an important marker in preventing possible complications and managing diagnosis and treatment. In our study, the rates of positive total β-hCG measurements in pregnant women with an additional disease were higher than in the group without.

In the management of ectopic pregnancies treated with various doses of methotrexate^22^-^24^ and in inducing abortion with misoprostol,^25^ β-hCG levels were quite useful. In our study, total β-hCG measurements were higher in the group that used the medication regularly than in those that did not use it and in the group that had pregnancy risk factors than in those that did not have risk factors. In one study, a rapid decrease was observed in serial hCG measurements taken in the first days after early medicated abortion until the 5th day.

Further studies are needed to determine how early this decrease is specific in terms of confirming that the abortion is complete.^26^ Another study reported that the serum/abortion material hCG ratio provided 91.7% accuracy within the first few hours to distinguish early pregnancy from miscarriage. It is emphasized that it is a reliable method for distinguishing ectopic pregnancy from miscarriage.^27^ One study reported that retained pregnancy material may occur after delivery or abortion and that operative hysteroscopy is often required for its removal, but it was stated that the β-hCG level did not contribute to this preoperative diagnosis.^28^ In our study, total β-hCG levels were higher in groups with pregnancy risk factors and complications than in groups without. In addition, β-hCG levels were higher in groups with abortion than in groups without.

In our study, important results were obtained as a result of the evaluation of β-hCG levels in pregnant women in our region, which should be decided in normal and abnormal situations. We emphasize that family physicians should approach pregnant women holistically and cooperate with obstetrics, gynecology, and other clinics, as β-hCG levels in the blood may lead to different results and abnormal processes due to different reasons. There is a need to share the results of β-hCG levels in the blood in different situations in the literature.

### Limitations:

Since our study is retrospective, the possibility that some of the results of consultations at other clinics were not included in our records may have created a limitation. Additionally, the follow-up and results of our pregnant women who left our region or migrated abroad may have also created limitations.

## CONCLUSION

In our study, blood β-hCG levels were higher in pregnant women who had an additional disease, those who were on medication, those who had risk factors and those who developed complications. Total β-hCG measurement levels were lower in those who had normal or cesarean live births, while they were higher in those who had abortions. Different results in blood β-hCG levels require a holistic approach to evaluate pregnancy. In this critical process, the collaboration of family physicians with obstetrics, gynecology and other clinics is important.
